# Eating Out-of-Home in Adult Residents in Shanghai and the Nutritional Differences among Dining Places

**DOI:** 10.3390/nu10070951

**Published:** 2018-07-23

**Authors:** Jiajie Zang, Baozhang Luo, Yaping Wang, Zhenni Zhu, Zhengyuan Wang, Xin He, Wenjing Wang, Yan Guo, Xiao Chen, Chunfang Wang, Changyi Guo, Shurong Zou, Xiaodong Jia, Fan Wu

**Affiliations:** 1 Department of Nutrition Hygiene, Division of Health Risk Factor Monitoring and Control, Shanghai Municipal Center for Disease Control and Prevention, Shanghai 200336, China; zangjiajie@scdc.sh.cn (J.Z.); wyp20170902@126.com (Y.W.); zhuzhenni@scdc.sh.cn (Z.Z.); wangzhengyuan@scdc.sh.cn (Z.W.); guochangyi@scdc.sh.cn (C.G.); zoushurong@scdc.sh.cn (S.Z.); jiaxiaodong@scdc.sh.cn (X.J.); 2 Department of Food Hygiene, Division of Health Risk Factor Monitoring and Control, Shanghai Municipal Center for Disease Control and Prevention, Shanghai 200336, China; luobaozhang@scdc.sh.cn; 3 Department of Molecular Biology for Public Health, Division of Non-communicable Diseases, Shanghai Municipal Center for Disease Control and Prevention, Shanghai 200336, China; hexin@scdc.sh.cn (X.H.); wangwenjing@scdc.sh.cn (W.W.); 4 Putuo Center for Disease Control and Prevention, Shanghai 200333, China; gqsh813@163.com (Y.G.); putuocx@126.com (X.C.); 5 Department of Vital Statistics, Division of Health Information, Shanghai Municipal Center for Disease Control and Prevention, Shanghai 200336, China; wangchunfang@scdc.sh.cn

**Keywords:** out-of-home eating, dietary intake, restaurants, canteens, Shanghai China

## Abstract

Background: With the rapid development of Shanghai’s economy, diet habits have undergone great changes. The study aimed to examine the situation of out-of-home (OH) eating in Shanghai adults and the nutrition characteristics of eating in different dining places, and to assess the social demographic determinants of eating OH. Method: Data was sourced from the Shanghai Diet and Health Survey (SDHS) involving people aged 18 years or older in 2012–2013. The food frequency questionnaire (FFQ) and three-day 24-h dietary recall (24-HDR) were used to collect dietary intake data on how people eat out in a cross-sectional study of 1689 adults. OH food refers to the food prepared or consumed away from home. We define that people who eat at least one meal prepared away from home in each survey have a habit of eating outside. The multiple linear and logistic regression methods were used for statistical analysis. Results: The prevalence of eating OH and at restaurants was only 55.1% and 31.8%, respectively. There was an increase in energy, protein, carbohydrate, fat, and iron intake while eating OH. Restaurant and company/school canteen consumption were both associated with an increase in daily total energy intake of 140 kcal and 91 kcal, and fat intake of 6.0 g and 4.3 g, respectively. However, eating at restaurants was associated with higher intake of 548 mg of sodium. However, no significant association was observed between eating at canteens and higher sodium intake. Conclusions: Eating OH related to a poor diet quality, and the diet quality was different between restaurant and canteen food. There may be a need for interventions to target residents’ overall dining-out behavior, particularly focusing on the consumption of restaurant food.

## 1. Introduction

The food culture of the world has undergone great changes in the past few decades. More and more people choose to dine out [1,2]. A recent survey in the United States showed that over 50% of adults reported eating out three or more times a week and more than 35% of adults reported eating fast-food meals at least twice weekly [3]. In China, the report on the status of residents’ nutrition and chronic diseases pointed out, in 2012, the proportion of residents aged 6 years and over in China dining out was 35.5%, that of urban residents dining out was 42.2%, and that of rural residents dining out was 28.5% [4].

As the number of eating out occasions continues to increase, people are increasingly paying attention to the quality of out-of-home (OH) meals. Homemade meals were considered to be healthier than out-of-home purchased meals [5]. Certain studies have indicated that eating out of home may increase the consumption of meat, sweets, and alcohol while reducing the intake of grains, vegetables, and fruits [6,7,8,9,10]. OH meals tend to be higher in energy, fat, salt, and sugar and lower in fiber, vitamins, and minerals than meals prepared at home [11,12,13,14]. Kant et al. found that weekly OH meal consumption was an independent, inverse correlate with the serum concentrations of vitamins D, E, vitamin B-12, folate, and α-carotene in women and in people ≥50 years old [3]. These studies have demonstrated that OH eating was associated with a poorer diet quality.

However, the findings of some studies were conflicting with most studies [15]. Lachat et al. found that eating outside was positively associated with dietary diversity and better dietary intake in terms of energy supply from fat in Vietnam. In rural areas, eating OH meals was positively associated with iron, fruit, meat, poultry, and offal intake [16]. Similar findings were found in two studies in Kenya. In low–middle-income areas, men who dine out regularly consume more iron and vitamin A than meals prepared at home [17]. OH foods were most likely to be fruits in rural areas. OH food provided substantial amounts of vitamin C and A, Ca, and folate during the harvest season. It was considered that eating outside can improve healthy diet options in some circumstances. The components of the food eaten OH is a key determinant of the dietary intake.

Although several studies reported that frequent OH eating was associated with higher daily energy intake and poorer diet quality [9,18,19,20], few studies have considered whether these associations differ or not according to dining places. A previous study [21] among young adults in the Minneapolis/St Paul metropolitan area of Minnesota reported that dining at fast-food restaurants was relevant to higher intake of total energy, sugar-sweetened beverages, and fat where primarily burgers and French fries are served. Moreover, it was associated with a lower intake of healthful foods and key nutrients. However, full-service restaurants, which were more frequently used, was related to higher intake of vegetables. Besides, studies among non-Hispanic black and non-Hispanic white adolescents and young American adults suggested that residents in communities with greater access to full-service restaurants consumed more fruits and vegetables [22,23], and were more likely to meet dietary recommendations for saturated fat [24,25]. Previous research has only focused on OH eating but has not distinguished between differences in dietary nutrient intake in different external food consumption settings. The characteristics of eating outside in different dining places are not clear yet. Determining if the use of specific away-from-home food establishments contributes to overall diet quality is significant for developing interventions to improve diet [26].

Since the reform and opening up in 1978, Shanghai has made great progress in the development of its society and economy. With the change of food supply system and the introduction of fast food, the consumption patterns and eating behavior of Shanghai residents have undergone great changes [27,28,29]. Meanwhile, in the past 30 years, the restaurant industry in Shanghai has been continued rapid growth. In 2014, mass catering, which mainly including popular restaurants, fast food, group meals, special dinners, local snacks, takeout meals, and street gear, accounted for 80% of the entire catering industry in Shanghai [30]. According to statistics released by the Shanghai Bureau of Statistics, Shanghai’s catering revenue in 2016 was 107.24 billion yuan, an increase of 4.7% year-on-year [31]. Dining out is increasingly common, especially in the young and middle-aged population [32]. Besides, unlike other foreign countries, it is common for occupational groups and students to dine in the company/school canteen in Shanghai.

Therefore, Shanghai, as the fastest growing city in China, has a development model that is the forerunner of the country. Under these circumstances, studying the situation of Shanghai residents dining out works to clarify the changes of people’s diet in the nutrition transit mode with Chinese characteristics, and provides the basis for other areas with the same characteristics. In order to understand public health implications, it is a necessity to monitor OH dietary choices.

The purpose of the present study was to evaluate the situation of overall OH eating in Shanghai adults, and to examine the nutrition characteristics of eating in different dining places, and to assess the social demographic determinants of eating OH.

## 2. Materials and Methods

### 2.1. Study Design

Data came from the Shanghai Diet and Health Survey (SDHS) of people aged 18 years or older from 2012–2013. The SDHS is an ongoing open cohort performed since 2012. It was designed and implemented by the Chinese government to examine the nutritional status and food contaminants in Shanghai and assess how they have an effect on human health. With the rapid economic growth in Shanghai, the consumption patterns and eating habits of Shanghai residents have undergone great changes, which may influence food intake and health outcomes. The specific sampling method is listed in the published literature [33]. A total of 1944 subjects (≥18 years old) and their family members were recruited. After excluding observations lacking information, finally, analysis relied on 1689 individuals, 842 men and 847 women.

The SDHS was approved by the Ethical Review Committee of Shanghai Municipal Center for Disease Control and Prevention. All participants were fully informed of the purpose and procedures of the study before enrolling and signed written consent forms.

### 2.2. The Definition of Eating Outside

Each subject was interviewed about his/her food consumption of the past week using the food frequency questionnaire (FFQ). Participants were asked “How many days have you had breakfast, lunch, or dinner in the past week (seven days)?” and subsequently “How many days in the past week have you had breakfast, lunch, or dinner at a restaurant or work (school) canteen?” In the present study, eating OH food refers to the food prepared or consumed away from home. We define that people who eat at least one meal prepared away from home in each survey have a habit of eating outside. Among them, we also define that, people who eat a meal or more prepared at restaurants have a habit of eating at restaurants, and people who consume a meal or more prepared at company/school canteens except restaurant have a habit of eating at company/school canteens. Those who do not meet the above conditions are defined as those who eat at home only. All the analysis based on the data from the whole cohort.

### 2.3. Dietary Data Collection

In the SDHS, dietary assessment was based on a combination of data collected at the individual level, with food inventory obtained at the household level. Individual dietary data was collected by the three-day 24-h dietary recall (24-HDR) in four separate visits, including the types and amounts of food, and food location. Every household member was asked to report all food consumed over the previous 24 h for three days (including two working days and one weekend day) whether at home or not. Household consumption of condiments (e.g., edible oils, salt, and sauces) was determined by weighing all food consumed by the household over three consecutive days. Three-day 24-HDR was performed on three consecutive days to match the weighing. All purchases and wasted condiments were also recorded. At the end of the survey, all remaining condiments were weighed again. The specific operation process is available in the published literature [33].

The food codes in the SDHS correspond to food names in the Chinese Food Composition Table and were used for food group classification. Total intake (in grams) for each food group was determined. Cooking oil and salt intake from household food consumption data were used to supplement individual dietary data. Individual cooking oil and salt consumption was calculated according to the total amounts of oil and salt consumed in the household divided by the proportions of energy consumption of each individual in the household.

### 2.4. Assessment of Sociodemographic Determinants

Age groups were divided into three categories (18–44, 45–59, and ≥60 years). Marital status was divided into two categories (married and other marital status) based on five categories in the questionnaire. Occupation status was grouped into three levels (professional job, labor job, and others). Years of education level was distributed into four categories, including ≤6 years, 7–9 years, 10–12 years, and >12 years. Each of the smoking and drinking statuses were defined as two levels (No/Yes). Body mass index (BMI) was divided into four categorical levels based on criteria recommended by the Working Group on Obesity in China, which was defined as (weight in kilograms)/(height in meters)^2^: underweight (<18.5 kg/m^2^), normal (18.5–23.9 kg/m^2^), overweight (24.0–27.9 kg/m^2^), and obese (>28.0 kg/m^2^). Height and weight were measured directly by trained health workers, as recommended by the World Health Organization. Participants were divided into three residency groups according to location (urban, suburban, and rural). Household size reported by subjects was divided into three categories (1–2 persons, 3 persons, and ≥4 persons). Family income was classified into four levels (<20,000 RMB/person, 20,000–50,000 RMB/person, >50,000 RMB/person, and not reported income).

### 2.5. Statistical Analysis

The frequency of eating OH is expressed as a percentage. Median and interquartile range were used to assess nutrient intake when eating outside, as these data were non-normally distributed parameters. The linear regression model was used to assess the differences of energy and nutrients intake between people eating outside or at home. The univariable and multivariable logistic regression models were employed to evaluate social demographic determinants of eating outside. *p* < 0.05 was considered statistically significant. The statistical/data analysis software package SAS 9.4 (SAS, Cary, NC, USA) was used for all data analysis.

## 3. Results

### 3.1. Characteristic of Study Participants

Table 1 shows the characteristics of Shanghai adult residents including mostly basic information and lifestyles. In this study, the actual number of people involved in the survey was 1689, of which 842 were adult males and 847 females, and the number of urban, suburban, and rural residents accounted for 49.02%, 24.33%, and 26.47%, respectively. The three age groups (18–44, 45–59, ≥60) accounted for 30.61%, 33.98%, and 35.41%, respectively. Married people accounted for 77.86%. The number of respondents with different education levels and family sizes is more evenly distributed. Smokers and drinkers accounted for 75.05% and 18.83%, respectively. The specific distributions of the marital status, education level, occupation, family income, smoking and drinking, residency, and weight status across different genders are presented in Table 1.

### 3.2. The Frequency of Eating Outside

Table 2 shows the respondents’ consumption of meals prepared out-of-home and only at restaurant during four surveys, respectively. The frequency of eating OH in Shanghai was 55.05%. The frequency of eating breakfast, lunch, and dinner outside was 25.60%, 47.03%, and 18.46% respectively. The proportion of eating lunch outside was highest, followed by breakfast, while the least for dinner. The frequency of eating OH of urban, suburban, and rural residents were 56.02%, 56.01%, and 48.14%, respectively. The distribution of three meals prepared away from home in urban, suburban, and rural area was similar to the overall distribution. People in rural areas are more likely to prepare food at home, compared with urban and suburban residents.

The frequency of eating at restaurants was 31.79%. The frequency of breakfast, lunch, and dinner in the restaurants was 17.47%, 12.79%, and 10.38%, respectively. The number of people who consumed at the restaurants to eat breakfast was the largest, while the least for dinner. The frequency of eating at restaurants of urban, suburban, and rural residents was 34.15%, 31.49%, and 17.85%, respectively. Urban dwellers eat out more often, compared with suburban and rural residents.

### 3.3. The Location of Eating Outside

**Figure 1:**
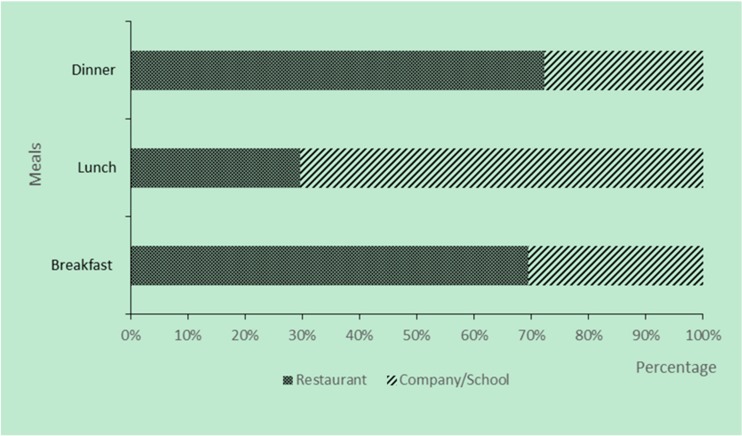
Location of eating outside.

In this survey, the location of eating outside mainly includes restaurants and company or school canteens. Figure 1 shows the composition ratio of the location of eating outside. The proportion of people who consumed breakfast, lunch, and dinner in the restaurant accounted for 69.49%, 29.68%, and 72.33%, respectively. The number of people who chose to eat breakfast at restaurants is the largest, while the lunch was more in the company or school canteens to eat.

### 3.4. Energy and Nutrients between People Eating at Restaurants or at Company/School Canteens

Table 3 shows the energy and nutrient intake between Shanghai residents eating at restaurants or at company/school canteens. Whether people consumed in a restaurant or in a company/school canteen, there was an increase in dietary energy, protein, carbohydrate, fat, and iron intake. Restaurant and company/school canteen consumption were associated with an increase in daily total energy intake of 140 kcal and 91 kcal, and fat intake of 6.0 g and 4.3 g, respectively. Moreover, those who consumed in the restaurants related to an increase intake with 0.1 mg of vitamin B1, and 0.8 mg of zinc. However, eating at restaurants was associated with higher intake of sodium. One meal consumed at restaurants was associated with an increase in daily sodium intake of 548 mg. However, no significant association was observed between eating at company/school canteens and at home.

### 3.5. Influential Factors of Eating Outside, Classified by Predictors

As shown in Table 4, the study reported a positive association of the behavior of eating outside and higher socioeconomic status. Adults from higher socioeconomic status, as measured by higher household income, higher education status, or urban or higher income residence were more likely to eat out (all ***p*** < 0.0001). In addition, the study suggests that males consume OH foods more frequently than women. The possibility of people who are not married eating outside was almost twice as much as married (OR: 1.84; 95% CI (1.44, 2.35)) (*p* < 0.0001). However, age, education levels, region, and drinking were significantly associated with the behavior of eating outside after adjusting. Young people and urban residents were relatively more likely to eat out of home. Higher education status had a higher possibility to consume at OH. Drinkers ate outside twice as likely as nondrinkers (OR: 2.23; 95% CI (1.56, 3.19)) (*p* < 0.0001).

## 4. Discussion

With rapid economic development, the dietary structure and lifestyle of the modern citizen have changed greatly. The study found that more than half of Shanghai residents ate out. Of the people who ate out, 70% of them were more likely to eat lunch in company/school canteens, and 69% and 72% of residents were more likely to eat breakfast and dinner in restaurants. There were differences in the dietary intake of different dining places.

We found that the frequency of eating meals prepared away from home in Shanghai during 2012–2013 was 55.05%, which was higher than the national average of 28.3% as reported by the China National Health and Nutrition Survey in 2002 and was 20% higher than the national average in 2012. A recent survey in the United States showed that over 50% of adults reported eating outside three or more times a week and more than 35% of adults reported eating fast-food meals at least twice weekly [3]. In the UK, more than one quarter (27.1%) of adults and one fifth (19.0%) of children ate meals out once per week or more. One fifth of adults (21.1%) and children (21.0%) ate take-away meals at home once per week or more during 2008–2012 [34]. There are many reasons for the difference in various studies. First, Shanghai’s overall economy has been in a period of rapid development and the level of urbanization in Shanghai is much higher than the national average in 2012 [35]. Rapid economic development may affect the level of residents’ consumption, purchasing power, and dietary patterns. Besides, in recent years, Shanghai’s catering industry has developed rapidly, in 2016, an increase of 4.7% year-on-year [31], which may be responsible for residents eating outside more frequently, compared with the country. Second, the differences in research results may be due to different research design. There is a lack of uniform standard for the definition of eating outside at present. Studies in the U.S. and U.K. mainly define the frequency of eating out, namely the number of times people consume foods at restaurants, fast food restaurants, coffee shops, supermarkets, etc., outside the home, including take-away consumption. However, the definition for our research is mainly based on the places of eating out, while places of dining out only refer to restaurants and company/school canteens, not including take-away food, and the scope is relatively small, which may underestimate the residents’ frequency of dining out. Moreover, the catering industry has continued to develop and transform, and the internet is bringing new growth to the traditional catering industry. According to the data from the China Catering Report (White Paper 2017), the growth of Internet catering in 2016 was as high as 300%, and the total turnover of the take-out industry was over 150 billion, accounting for 4% of the overall market for the catering industry [36]. Now Shanghai residents may eat out more frequently than they did in 2012, and the frequency of eating outside in Shanghai may be higher than that of the US and UK. At present, our research data are mainly from the results of the 2012–2013 survey. With the continuous development of the catering industry, the convenience of online payment, the continuous emergence of online take-away food service platforms, and the frequency of dining out of Shanghai residents will probably present a growing trend. Therefore, it is suggested that there is a need for interventions to strengthen the monitoring of the situation of residents dining out.

The study showed that, in general, OH eating was associated with an increase in daily intake of energy, fat, and sodium among adult residents in Shanghai, compared with eating at home. This is consistent with the findings of R. An [37] and Chorong Oh et al. [38]. Moreover, eating at a company/school canteen reported a relatively smaller contribution to daily intake of energy, protein, carbohydrates, and fat, compared to eating at restaurants. Eating at restaurants was significantly related to higher intake of dietary sodium, and no significant association was observed between eating at company/school canteens and higher dietary sodium intake. Restaurants’ diet quality was worse than that of canteen. The results of the current study are consistent with one previous study that total energy intake was higher among eaters who reported eating at restaurants but not among the eaters who reported eating only at work [39]. This is an interesting finding. It suggested that the intake of nutrients may differ according to dining place type. When eating in the restaurant, in order to better improve the taste, color, and flavor of the food, artificially add too much cooking oil, sodium, sugar, and use some unhealthy cooking methods such as frying, etc. [40]. As a result, foods produced and sold in restaurants and other places are high in energy density, saturated fat, added sugar, and sodium^1^. Studies suggested that, on average, meals from sit-down restaurants contained more than a full day’s worth of sodium and nearly a day’s worth of fat and saturated fat. The quality of dietary is poor when consuming at restaurants [7].

However, in China, relevant government departments pay more attention to the dietary quality of the unit/school canteens. There are agencies that engage in nutritional dietary interventions, such as the promotion of nutrition and health knowledge and nutrition training for employees, so that meals prepared at canteens have better quality than restaurants [41]; it was largely responsible for the differences of dietary nutrient intake in restaurants and canteens. This also provides direction for the next step in the development of public health strategies. It is not just to regulate the canteen’s diet, but also to focus more on the quality of the restaurant’s diet. In particular, there is a need for health behavior messages to address that meals prepared away from home, especially restaurants, are high in fat and sodium. The results of this study and others suggest that effective efforts are needed to encourage residents to alternatively prepare food at home when possible and provide tools to help in selecting healthy options when eating away from home [21,42,43]. For example, providing accurate, clear, and consistent nutrition information, including the calorie content of foods, in restaurants and similar retail food establishments will make such nutrition information available to consumers in a direct and accessible manner to enable consumers to make informed and healthful dietary choices [44]. Research shows that the impact of accurate nutrition labeling relates to the overall improvement of consumer health through more informed food choices and there is an opportunity to reduce the enormous economic costs that are associated with poor food choices in the process [45].

The main influencing factors for eating outside for adult residents in Shanghai were age, education level, region, and drinking status. High socioeconomic status and urban residence were associated with higher consumption of away-from-home meals. These are consistent with the findings of previous research that men, unmarried, highly educated, and high-income people were more likely to choose to eat out [1,46,47,48]. With the increase of education level, the probability of residents eating outside was higher, which may be due to the time saving and convenience of dining out. The closer people are to the center of the city, the more they tend to eat outside, possibly because of the rapid development of the catering industry and intensive distribution of restaurants in urban centers, and people have easy access to food prepared away from home. One study showed that [49] for every standard deviation increase in fast-food exposure, the odds of consuming fast food near home increased by 11–61% and the odds of a healthy diet decreased 3–17%. High socioeconomic status was related to higher consumption of away from home meals, and a study of the same population [33] found that the diet was of good quality, suggesting that people with higher socioeconomic status have better health literacy and tend to choose healthy food even if they are eating out.

The data of this study are from field surveys conducted in the four quarters of 2012–2013, with a large sample size and representative sample. It is significant that for the first time we have carried out a survey on the OH eating habits of Shanghai residents, examining the relationship between eating OH and the daily food intake of participants, and assessed the social demographic determinants of eating OH. In China, people pay more attention to the quality of family meals and launched corresponding improvement measures. There are few researches on the dietary status of residents dining out. Past researches agreed that health was closely related with diet. Out of home eating has become an integral part of the daily lives of residents. Dining quality and health issues for eating out are also becoming increasingly prominent. The results of this study can provide reference for other countries or regions with similar situations. Besides, we also found that dietary nutrient intake was different between dining places. This also suggests that the focus of public health interventions to improve diet quality should be different.

The present study had several limitations, primarily because of the definition of eating OH, which do not include take-out foods. It is possible to underestimate the frequency of dining out. We also recognize that our findings should be generalized with caution to the whole Chinese population, as differences in health awareness, socioeconomic status, and lifestyle might differ significantly between the general population and the current study population.

In conclusion, the results of this study demonstrated that more than half of Shanghai adult residents are eating out. Whether eating in a restaurant, or consuming food in a school/company canteen, both were related to excessive intake of energy and fat, and in general, the food prepared at restaurants had a poorer diet quality than food prepared at canteens. Eating outside is an important nutritional issue in Shanghai, China. Therefore, a comprehensive policy intervention is warranted to target residents’ overall dining-out behavior, particularly focusing on the consumption of restaurant food.

## Figures and Tables

**Table 1:** Characteristics of study participants.

Characteristics	Men	% of Sub-Group	Women	% of Sub-Group	All	% of Sub-Group
	*n*		*n*		*n*	
Age group (years)						
18–44	261	31.0	256	30.2	517	30.6
45–59	282	34.5	292	34.5	574	34.0
≥60	299	35.5	299	35.3	598	35.4
Marital Status						
Married	672	79.8	643	75.9	1315	77.9
Other marital status	170	20.2	204	24.1	374	22.1
Occupation						
Professional job	218	25.9	146	17.2	364	21.6
Labor job	103	12.2	75	8.9	178	10.5
Others	521	61.9	626	73.9	1147	67.9
Years of education (years)						
≤6	174	20.7	231	27.3	405	24.0
7–9	234	27.8	250	29.5	484	28.7
10–12	216	25.7	191	22.6	407	24.1
>12	218	25.9	175	20.7	393	23.3
Weight Status						
Underweight	28	3.3	32	3.8	60	3.6
Normal	403	47.9	492	58.1	895	53.0
Overweight	322	38.2	260	30.7	582	34.5
Obese	80	9.5	35	4.1	115	6.8
Non-measured	9	1.1	28	3.3	37	2.2
Smoker						
No	427	50.7	841	99.29	1268	75.1
Yes	415	49.3	6	0.71	421	25.0
Drinker						
No	506	60.1	778	91.85	1284	76.0
Yes	275	32.7	43	5.08	318	18.8
Non-reported	61	7.2	26	3.07	87	5.2
Household size						
1–2 persons	382	45.4	356	42.03	738	43.7
3 persons	295	35.0	317	37.43	612	36.2
≥4 persons	165	19.6	174	20.54	339	20.1
Family Income (RMB/person)						
<20,000	289	34.3	316	37.31	605	35.8
20,000–50,000	428	50.8	416	49.11	844	50.0
>50,000	125	14.9	114	13.46	239	14.2
Non-reported	0	0.00	1	0.12	1	0.1
Region						
Urban	412	48.9	419	49.47	831	49.2
Suburban	204	24.2	207	24.44	411	24.3
Rural	226	26.8	221	26.09	447	26.5

**Table 2:** Frequency of eating OH and restaurant meals of adults living in urban, suburban, and rural areas.

Item	Meals	All	% of Sub-Group	Urban	% of Sub-Group	Suburban	% of Sub-Group	Rural	% of Sub-Group
		*n*		*n*		*n*		*n*	
Eating OH	Breakfast	414	25.6	237	26.7	93	24.9	84	19.6
	Lunch	841	47.0	422	46.7	202	51.7	217	44.3
	Dinner	334	18.5	158	17.6	69	19.8	107	22.4
	One Day	942	55.1	492	56.0	215	56.0	235	48.1
Eating at restaurants	Breakfast	263	17.5	172	19.1	59	17.1	32	7.9
	Lunch	192	12.8	127	13.2	34	16.2	31	7.0
	Dinner	154	10.4	100	10.9	28	12.3	26	5.6
	One Day	466	31.8	299	34.2	92	31.5	75	17.9

**Table 3:** Energy and nutrients between people eating outside or at home.

Items	β1 (Restaurant)	t1	P1	β2 (Canteen)	t2	P2
DQD	0.44	0.85	0.397	0.42	0.83	0.407
HBS	**0.62**	**2.97**	**0.003**	**0.64**	**3.13**	**0.002**
LBS	−0.117	−0.34	0.732	−0.28	−0.58	0.559
Energy (kcal)	**139.71**	**3.58**	**0.000**	**90.55**	**2.37**	**0.018**
Protein (g)	**5.98**	**2.80**	**0.005**	**4.40**	**2.10**	**0.036**
Carbohydrate (g)	**14.95**	**2.94**	**0.003**	**7.72**	**1.55**	**0.122**
Fat (g)	**6.05**	**2.93**	**0.003**	**4.30**	**2.13**	**0.034**
Vitamin A (μg)	48.29	1.15	0.252	66.95	1.62	0.105
Vitamin D (μg)	−0.46	−0.33	0.743	−2.01	−1.48	0.140
Vitamin B1 (mg)	**0.08**	**4.04**	**<0.000**	0.03	1.65	0.100
Vitamin B2 (mg)	0.05	1.71	0.087	0.03	1.18	0.238
Vitamin B6 (mg)	−0.00	−0.78	0.436	−0.00	−0.97	0.334
Vitamin B12 (μg)	0.01	0.84	0.400	−0.01	−0.51	0.609
Vitamin C (mg)	2.26	0.50	0.619	−0.58	−0.13	0.897
Na (mg)	**547.74**	**2.39**	**0.017**	220.34	0.98	0.326
Ca (mg)	15.93	0.78	0.437	6.35	0.32	0.752
Zn (mg)	**0.78**	**2.75**	**0.006**	0.48	1.75	0.080
Fe (mg)	**2.16**	**2.76**	**0.006**	**1.50**	**1.96**	**0.050**

Note: Multivariable Model were adjusted by gender, age, marriage status, occupation, smoking, drinking, BMI status, residency, number of family members, and income. DQD: diet quality distance; HBS: higher bound score; LBS: lower bound score. Values in bold: statistical significant compared to that at home.

**Table 4:** Logistic regression models for eating outside, classified by predictors.

Items	Univariable Model		Multivariable Model	
	OR (95% CI)	*p*-Value	OR (95% CI)	*p-*Value
**Sex**				
Men	Reference		Reference	
Women	**0.61 (0.49, 0.75)**	**<0.000**	0.80 (0.56, 1.13)	0.060
**Age group (years)**				
18–44	Reference		Reference	
45–59	**0.44 (0.34, 0.57)**	**<0.000**	**0.47 (0.33, 0.67)**	**<0.000**
>60	**0.17 (0.13, 0.23)**	**<0.000**	**0.24 (0.16, 0.36)**	**<0.000**
**Marital Status**				
Married	Reference		Reference	
Other marital status	**1.84 (1.44, 2.35)**	**<0.000**	1.27 (0.88, 1.84)	0.204
**Occupation**				
Professional job	Reference		Reference	
Labor job	**0.35 (0.23, 0.52)**	**<0.000**	0.73 (0.41, 1.30)	0.284
Others	**0.36 (0.28, 0.46)**	**<0.000**	0.75 (0.53, 1.07)	0.117
**Years of education (years)**				
≤6	Reference		Reference	
7~9	**3.90 (2.53, 6.02)**	**<0.000**	**2.14 (1.25, 3.67)**	**0.006**
10~12	**6.29 (4.09, 9.68)**	**<0.000**	**3.36 (1.94, 5.81)**	**<0.000**
>12	**11.71 (7.64, 17.94)**	**<0.000**	**4.83 (2.73, 8.55)**	**<0.000**
**Smoker**				
No	Reference		Reference	
Yes	**1.57 (1.23, 1.99)**	**0.000**	1.12 (0.75, 1.66)	0.574
**Drinker**				
No	Reference		Reference	
Yes	**2.02 (1.56, 2.62)**	**<.0000**	**2.23 (1.56, 3.19)**	**<0.000**
**Weight Status**				
Underweight	0.81 (0.44, 1.52)	0.515	0.63 (0.30, 1.32)	0.223
Normal	Reference		Reference	
Overweight	0.82 (0.63, 1.07)	0.145	0.94 (0.69, 1.29)	0.716
Obese	0.99 (0.62, 1.59)	0.969	1.30 (0.73, 2.31)	0.369
**Region**				
Urban	Reference		Reference	
Suburban	**0.53 (0.40, 0.69)**	**<0.000**	**0.53 (0.37, 0.76)**	**0.001**
Rural	**0.36 (0.27, 0.48)**	**<0.000**	**0.39 (0.27, 0.57)**	**<0.000**
**Household size**				
1-2 persons	Reference		Reference	
3 persons	**1.37 (1.08, 1.74)**	**0.011**	0.75 (0.55, 1.05)	0.079
≥4 persons	1.17 (0.88, 1.57)	0.287	1.28 (0.87, 1.88)	0.282
**Family Income (RMB/person)**				
<20,000	Reference		Reference	
20,000–50,000	**1.70 (1.33, 2.17)**	**<0.000**	1.24 (0.90, 1.72)	0.192
>50,000	**1.97 (1.41, 2.75)**	**<0.000**	1.17 (0.75, 1.85)	0.492

Values in bold: statistical significant compared to references.
